# A secreted Heat shock protein 90 of *Trichomonas vaginalis*

**DOI:** 10.1371/journal.pntd.0006493

**Published:** 2018-05-16

**Authors:** Meetali Singh, Divya Beri, Rishi Kumar Nageshan, Leena Chavaan, Darshak Gadara, Mukta Poojary, Suraj Subramaniam, Utpal Tatu

**Affiliations:** Department of Biochemistry, Indian Institute of Science, Bangalore, India; National University of Singapore, SINGAPORE

## Abstract

*Trichomonas vaginalis* is a causative agent of Trichomoniasis, a leading non-viral sexually transmitted disease worldwide. In the current study, we show Heat shock protein 90 is essential for its growth. Upon genomic analysis of the parasite, it was found to possess seven ORFs which could potentially encode Hsp90 isoforms. We identified a cytosolic Hsp90 homolog, four homologs which can align to truncated cytosolic Hsp90 gene products along with two Grp94 homologs (ER isoform of Hsp90). However, both Grp94 orthologs lacked an ER retention motif. In cancer cells, it is very well established that Hsp90 is secreted and regulates key clients involved in metastases, migration, and invasion. Since *Trichomonas* Grp94 lacks ER retention motif, we examined the possibility of its secretion. By using cell biology and biochemical approaches we show that the Grp94 isoform of Hsp90 is secreted by the parasite by the classical ER-Golgi pathway. This is the first report of a genome encoded secreted Hsp90 in a clinically important parasitic protozoan.

## Author summary

Hsp90 is an essential chaperone in eukaryotes and it is often described as a master regulator of cellular homeostasis. In addition to its well-known functions inside the cell, extracellular Hsp90 has also been implicated in migration and invasion of tumor cells. We have, for the first time, identified the presence of an extracellular Hsp90 in a parasitic protozoan, *Trichomonas vaginalis*. The extracellular Hsp90 is a Grp94 homolog that lacks a canonical ER retention signal. Our analysis of Grp94 sequences from protozoa shows that it is uncommon for a Grp94 to lack ER retention signal. In the current study, we characterized the biochemical parameters and established the extracellular localization of this Hsp90 paralog. This secreted Hsp90 in *Trichomonas* can potentially modulate host-pathogen interaction.

## Introduction

*Trichomonas vaginalis* is the causative agent of Trichomoniasis, a leading non-viral sexually transmitted disease worldwide [1]. The pathogen induces local inflammation of the lower genitourinary tract, can be involved in premature labor and low birth weight. Trichomoniasis affects both males and females with higher prevalence in females in both developed and developing countries [1]. Epidemiological studies show a two to three-fold increase in the risk of HIV infection in people with Trichomoniasis due to HIV target cells, CD4+ T-lymphocytes, recruitment at the site of infection [2, 3]. This parasite infects more than 270 million people worldwide annually, despite which there is no clear consensus on its pathogenicity mechanisms. Due to the synergy with HIV infection and increase in reports of drug resistance, it is pivotal to understand the basic survival principles of the parasite [2, 4].

This extracellular parasite survives in a dynamic and physiologically diverse niche of urogenital tract and therefore, is likely to possess a robust stress-response machinery to persist and establish infection. Our studies on related parasitic protozoa show the important role of stress-regulated heat shock protein 90 in their life cycle and virulence [5–14]. When we analyzed Hsp90 sequences in *Trichomonas* genome, we found two Hsp90 isoforms (TVAG_030810 and TVAG_378910, henceforth referred to as TV810 and TV910 respectively), which lacked classical motif of either cytosolic Hsp90 or Grp94 [15]. We show that these two isoforms are Grp94 homologs, however, both lacked an ER retention signal. This observation was very peculiar since most of the studied Grp94s have the canonical ER retention signal. Therefore, we were intrigued about what could be the possible fate of *Trichomonas* Grp94 homologs. In absence of ER retention signal, one possibility is that these two Hsp90 orthologs are secreted. In cancer cells, it is very well established that Hsp90 is secreted and regulates key clients like matrix metalloproteinases (MMPs) which are involved in migration and invasion [16]. It is known to play an important role in epithelial to mesenchymal transition, thereby regulating tumor metastasis [16–20]. Majority of these studies have focused on studying the role of extracellular Hsp90 in context of cancer and there are not many well-documented studies on extracellular Hsp90 in other organisms including parasitic protozoa.

In the current study, we show that Hsp90 inhibition is lethal to *Trichomonas* growth and hence Hsp90 function is critical for the survival of the parasite. Using cell biology, biochemical and proteomics approaches, we investigated the localization of Grp94 ortholog, TV910, and found it to be secreted by *Trichomonas* in the culture medium by the classical ER-Golgi secretory pathway. This is the first report of a secreted Hsp90 in a clinically important parasitic protozoan.

## Materials and methods

### Antibodies

A polyclonal antibody was raised in New Zealand White strain rabbit against 6x-His-tagged full-length TVAG_378910 (TV910) recombinant protein expressed in *E*. *coli*. Dilutions of the antibodies used for different experiments: For Western blot: anti-TV910 at 1:500; For IP: anti-TV910 at 1:50. Anti- α-tubulin antibody, 12G10 produced in mouse (a kind gift from Dr. Carsten Janke, Institute Curie, Orsay) was used at the dilution of 1:500 for blot. HRP conjugated Goat anti-rabbit IgG and anti-mouse IgG antibodies (Bangalore Genie). 1:10000 dilution was used for both the antibodies.

### Ethics

Animals were maintained, and experiments were performed as per the principles, guidelines, and methods approved by the Institutional Animal Ethics Committee (IAEC) of the Central Animal Facility (CAF), Indian Institute of Science, Bangalore. The methods were approved by Committee for the Purpose of Control and Supervision of Experiments on Animals (CPCSEA), Ministry of Environment, Forest and Climate Change Welfare Division, Government of India. The committee approved the experiments performed (Project Number: CAF/Ethics/269/2012).

### List of primers used

TV560 Fwd: 5’-GGGGGGATCCATGTCTGCTGAAGTCGAAACACTTG-3’, TV560 Rev: 5’-GGGGGAATTCTTAATCGACATCATCAAACTTATTAAGG-3’, TV910 Fwd: 5’-GGGGGGATCCATGGACCTTCGTGAGAAGCTC-3’, TV910 Rev: 5’-GGGGCCATGGCTAAAGTATACCTGCATAACATTCTTC-3’, TV810 Fwd: 5’-GGGGGGATCCATGTTTCAAGTTATCTTTTTTGCAAAGG-3’, TV810 Rev: 5’-GGGGCCATGGTTAGGGTTCATTGACGTTTTCG-3’.

### Hsp90 isoforms in *Trichomonas vaginalis*

Hsp90 isoforms were identified in *Trichomonas* genome by BLASTp in trichdb.org. Multiple sequence alignment was carried out for all three full-length Hsp90 isoforms–TVAG_153560 (TV560), TVAG_378910, TVAG_030810 using MUSCLE algorithm at ebi.uk. For promoter analysis, motif search for Inr elements and M5 like elements was done for a region 400 bp upstream of transcription start site. Comparison of Hsp90 and Grp94 (XP_641313.1) canonical motifs was done with human Hsp90 (NP_005339.3) and Grp94 respectively.

For phylogenetic clustering of Hsp90 isoforms, maximum likelihood algorithm was used as described above. Sequences used were retrieved from NCBI and Eupathdb. Gene IDs used for cytosolic Hsp90s were *Saccharomyces cerevisiae* NP_013911.1, *Homo sapiens* NP_005339.3, *Entamoeba histolytica* XP_653132.1/ EHI_196940, *Cryptosporidium parva* XP_626924.1, *Babesia bovis* XP_001611554.1, *Plasmodium falciparum* XP_001348998.1, *Toxoplasma gondii* XP_002368278.1, *Trypanosoma brucei* A44983, *Giardia lamblia* BAJ33526.1, *Trichomonas vaginalis* TVAG_153560, *Neospora caninum* XP_003881046.1, *Mus musculus* NP_032328.2, *Gallus gallus* NP_996842.1, *Danio rerio* NP_571403.1, *Brugia malayi* EDP29326.1, *E*. *coli* HtpG EDV65681.1 and *Klebsiella* Htpg CCI78437.1. Gene IDs for Grp94s used for analysis were: *Entamoeba histolytica* (EHI_163480), *Cryptosporidium parva* (cgd7_3670), *Giardia lamblia* (DHA2_15247), *Plasmodium falciparum* (PF3D7_1222300), *Babesia bovis* (BBOV_IV008400), *Theileria annulata* (TA06470), *Toxoplasma gondii* (TGGT1_244560), *Neospora caninum* (NCLIV_019110), *Bos taurus* (NP_777125.1), *Mus musculus* (NP_035761.1), *Danio rerio* (NP_937853.1), *C*. *elegans* (NP_001255536.1).

### Analysis of Grp94s lacking ER retention motif

Hsp90 protein sequences were retrieved by performing a simple BLASTp search with default parameters against major phyla of Protista clade including various taxonomic clades of Protista including Alveolata, Amoebozoa, Apicomplexa, Ciliophora, Diplomonadida, Euglenozoa, Microsporidia, Myxospora, and Parabasalia. Protein BLASTp search was done with default parameters using Human Grp94 protein sequence (Accession number: AAH66656) and sequences were filtered on the basis of their Query coverage (>60%), Identity (>30%) and e-value (less than 0.01). Protein sequences fulfilling the above criteria were considered for further analysis. The sequences not having ER retention signal (KDEL motif or KDEL-like motifs) were then filtered using an in-house Perl script. Multiple Sequence alignment of sequences lacking canonical retention motifs and some known Hsp90 orthologs, was performed using MUSCLE algorithm from MEGA 7 suites, followed by the construction of a Maximum Likelihood tree with 1000 bootstraps. The sequences clustering along with Cytoplasmic or Mitochondrial Hsp90 sequences were excluded from further analysis. The Hsp90 sequences clustering with Human Grp94 sequence were then confirmed to be indeed Grp94 homolog by reverse BLAST analysis. Sequences yielding known Grp94 sequence as the topmost hit were identified as true Grp94 homologs which do not have a canonical KDEL or KDEL-like retention motif.

### Parasite culturing

*Trichomonas vaginalis* isolate (Strain is a kind gift from Prof. Daman Saluja and Dr. Manisha Yadav, ACBR, New Delhi) was cultured in glass tubes in TYI-S-33 medium at 37°C containing 10% heat-inactivated adult bovine serum (HiMedia), and 2.5% Diamond vitamin mix. *G*. *lamblia* Portland P1 parasites were cultured in TYI-S-33 [6] supplemented with 12% fetal bovine serum.

### RNA extraction and PCR

RNA extraction was carried out using TriZol reagent (Thermo Fisher Scientific) according to manufacturer’s instructions. The concentration and purity of the RNA extracted were evaluated using the Nanodrop spectrophotometer (Thermo Scientific; 1000). 2 μg RNA of all samples was used to synthesize cDNA using Verso cDNA Synthesis kit (Thermo Fisher Scientific) according to manufacturer’s instructions. Amplification for genes was performed with respective primers as listed in previous section in Mastercycler (Eppendorf). PCR products were analyzed on a 2% agarose gel with ethidium bromide.

### Cloning and purification of parasite Hsp90s

TV910 and TV560 were amplified from cDNAs of respective parasites using the primers. Amplified products were cloned in pRSET-A vector as a 6x-His tag fusion protein.

Both proteins were expressed in *E*. *coli* BL21 pLysS expression strain and proteins were purified to homogeneity using Ni-NTA affinity chromatography. In brief, *E*. *coli* strains of clones were grown in LB broth and induced with 0.2 mM IPTG at 16˚C for 8 hours. Cells were pelleted down and lysed in buffer containing Tris-Cl pH 7.5, 1 M NaCl and 10 mM imidazole and the protease inhibitors. Lysate supernatants were allowed to bind to Ni-NTA beads. Beads bound to proteins were then washed with buffer containing a gradient of imidazole concentration ranging from 10 mM to 50 mM. Proteins were finally eluted using a buffer containing 200 mM imidazole. Proteins were then dialyzed in suitable buffers described in following sections for different experiments. Protein concentration was estimated using Bradford’s reagent using BSA as standard.

### Cell lysis

*Trichomonas* trophozoites grown to log phase were harvested by chilling on ice for 5 min and centrifuging at 600 g and were lysed by repeated freeze-thaw in liquid nitrogen in PBS containing 0.1% Triton X-100 and protease inhibitors.

### Assay for parasite sensitivity to 17-AAG

*Trichomonas* trophozoites grown to log scale were harvested and 15,000 cells were seeded per well in TYI-S-33 medium in a 96-well plate. Cells were allowed to adhere. Medium was replaced with the medium containing varying concentrations of 17-AAG from 10 nM to 100 µM. 0.2% DMSO was used as the vehicle control. Cells were treated with the drug for 24 hours. Post-treatment, viable *Trichomonas* cells were counted under a microscope using trypan blue dye exclusion method. Percent survival above control was plotted against the log concentration of 17-AAG and data was analyzed using GraphPad Prism 5.0 and GI_50_ (50% Growth inhibitory concentration) was determined. Inhibition assay for extracellular Hsp90 was performed in a similar manner using Fluorescein isothiocyanate-Geldanamycin (FITC-GA; Enzo Lifesciences), which is a cell impermeable inhibitor of Hsp90 [21].

### ATP and 17-AAG binding to Hsp90s

ATP binding was determined using the method of fluorescence quenching upon ligand binding as described previously [8, 12]. Briefly, 50 µg protein in binding buffer (40 mM Tris-Cl buffer pH 7.4, 5 mM MgCl_2_ and 100 mM KCl) was incubated with different concentrations of ATP (0.05–10 mM). Intrinsic tryptophan fluorescence was measured by scanning the emission spectrum in the wavelength range of 300–400 nm and excitation at 280 nm. The fluorescence intensity at λ_max_ 340 nm was selected for calculations. The difference in intrinsic fluorescence of protein alone and in the presence of the ligand was plotted against the ligand concentration. Data was analyzed using GraphPad Prism 5.0 using non-linear regression analysis with single site-specific binding. A similar procedure was carried out for 17-AAG binding with 50 µg protein in binding buffer with the concentrations of 17-AAG ranging from 500 nM—50 μM. The final concentration of DMSO in the assay was 1%.

### ATPase assay for Hsp90s

1.5 μM of Hsp90 protein in 40 mM Tris-Cl buffer, pH 7.4 containing 100 mM KCl and 5 mM MgCl_2_ was incubated with varying concentrations of ATP (50 to 4000 μM). γ^32^P-ATP with specific activity of 0.55 Ci/mmole was used as a tracer. 300 µM of 17-AAG was used in the control reaction to negate out non-specific or background activity. Control activity was subtracted from the total activity. ATPase activity was plotted against the ATP concentrations. Data was analyzed using GraphPad Prism 5.0 using Michaelis-Menten kinetics.

### ATPase inhibition assay by 17-AAG

ATPase inhibition assay was carried out in a similar manner as described above, except that the purified Hsp90 protein was incubated with a saturating concentration of ATP (2 mM) and 17- AAG concentration was varied from 2.5 µM to 150 µM. 300 µM 17-AAG was used in the control reaction. Percentage residual ATPase activity was plotted against the log concentration of inhibitor and the result was analyzed using GraphPad Prism 5.0.

### Secretion assay

For secretion assay, log phase grown *Trichomonas* or *Giardia* trophozoites were pelleted at 600×g and washed thrice with PBS. Washed cells were resuspended at 10^5^ cells/mL density in PBS sucrose (5% w/v). Cells were incubated at 37°C for desired time. Following incubation, cells were harvested by centrifuging at 600×g for 10 min, spent media was filtered through 0.22 μm syringe filter and concentrated using Amicon Ultra filters. The sample was further processed for either SDS PAGE, 2DGE or IP.

### In-gel and in solution trypsin digestion

Peptides were extracted either by in-gel trypsin digestion from each gel piece, or the protein solution was processed for digestion in solution. Proteins were first reduced using 10 mM DTT and alkylated by 55 mM iodoacetamide followed by 16 hours in-gel trypsin digestion (20 ng/µL, Promega). Peptides were then extracted in 5% formic acid and 60% acetonitrile. Extracted peptides were dried under vacuum.

### LC-MS/MS analysis

Vacuum dried digested peptide mixtures were dissolved in the solvent (2% acetonitrile and 98% water containing 0.5% formic acid) and further fractionated by Reverse Phase chromatography on C-18 material on a nano- HPLC system connected online to a nanospray ESI hybrid Q-TOF mass spectrometer from Applied Biosystems. A 100 µm ID, 5 µm particle size, 100 Å porosity Michrom column was used for chromatographic separation. The RP chromatography runtime was set for 144 minutes with a flow rate of 300 nL/minute. The mobile phase was adjusted to an increasing concentration of acetonitrile from 5% to 90%, to elute peptides from the column based on their hydrophobicity.

Analyst QS software was used to systematically acquire the TOF MS and MS/MS data for each precursor ion entering the instrument from the nanoLC. Eluted peptides were analyzed by one full MS scan and four consecutive product ion scans of the four most intense peaks, using rolling Collision Energy and Dynamic Background Subtract function. An Information Dependent Acquisition (IDA) experiment was used to specify the criteria for selecting each parent ion for fragmentation which included selection of ions in m/z range: > 400 and <1600, of charge state of +2 to +5, exclusion of former target ions for 30 seconds, accumulation time of 1 second for a full scan and 2 seconds for MS/MS, ion source voltage of 2200 V. The original data files were analyzed using the Protein Pilot software from a combined database (Swiss Prot 2005, TrEMBL, NCBI, and PDB). Peptide scores above 50 and a protein score of minimum 1.3 corresponding to a confidence level greater than 95% were used.

We further developed an MRM (Multiple reaction monitoring) based detection method for TV910. The Skyline was used to generate peptides by *in silico* trypsin digestion and to predict their MRM. The length of the peptide was kept between 8–16 amino acids. The peptide with consecutive K and R (KR, KK, RR, and RK) were not considered to avoid miscleavage sites by trypsin. Peptides with cysteine and methionine were avoided due to their instability. Two peptides were selected, based on the previously mentioned criteria [22]. Their uniqueness was confirmed to *T*. *vaginalis* TV910 by BLASTp analysis. The selected peptides were DELINNLGGIAK and IENVILSK. Using pure TV910, MS response and retention time were determined. Optimized MRM transitions and chromatographic conditions of the selected peptides are described in S2 and S3 Tables.

Secretion assay was performed as described in the previous section and secreted proteins were then resolved on an SDS-PAGE. The band near ~ 90 kDa for blank and the lysate was excised and processed as mentioned above. The digested peptide mixtures were dissolved in the solvent (2% acetonitrile and 98% water containing 0.5% formic acid) and analysed on LC-MS/MS (Agilent 1260 Infinity LC coupled with 6460 Triple Quad MS). Gradient chromatographic separation was achieved on Agilent Eclipse C18 (4.6 ×250 mm, 5μm) column by using mobile phase A (0.1% formic acid in MilliQ water) and mobile phase B (0.1% formic acid in Acetonitrile. The run time was 32 min, at the flow rate of 0.4 ml/min. Data analysis was done by Agilent Mass Hunter Qualitative Analysis B.07.00.

### Brefeldin A treatment of *Trichomonas*

Cells were treated with BFA at a concentration of 10 μg/mL for 2 hours at 37°C at a cell density of 10^5^ cells/mL.

### S^35^ labeling, pulse-chase, and immunoprecipitation

*Trichomonas* cells were starved by incubating in Cys and serum-free medium for two hours. S^35^ met and cys radiolabel was added to the culture at 100 µCi/mL. Cells were incubated with the label for 2 hours and label was washed off by PBS washes. Chase for different time points or in presence or absence of BFA was carried out. Lysate and spent medium samples were prepared as described in previous sections.

Pre-clearing of samples was done by incubating 1/10^th^ volume of Protein A-agarose beads with samples at 4°C on an end-to-end rotor for 1.5 hours. After pre-clearing, beads were spun down and the supernatant was taken in a fresh tube, desired primary antibody was added to test samples and in Protein A control, the antibody was not added. Samples were incubated for 12 hours at 4°C on end to end rotor. After 12 hours 1/10^th^ volume of Protein A- agarose beads were added to samples and incubated for 3 hours at 4°C. After this step, Protein-A beads were spun down and were washed with 1 ml IP buffer (PBS with 0.1% Triton X-100) on vortex for 15 minutes each and four such washes were done. After washes, the beads alone were taken and suspended in 50 µL of Laemmli buffer and were boiled for 10 minutes and centrifuged at 20,000 rpm for 15 minutes. The supernatant was then resolved on an SDS- 10% Polyacrylamide gel. The gel was coomassie stained and dried. The dried gel was exposed on phosphor screen for 5 days and scanned by PhosphorImager.

## Results

### Hsp90 isoforms in *T*. *vaginalis*

**Fig 1:**
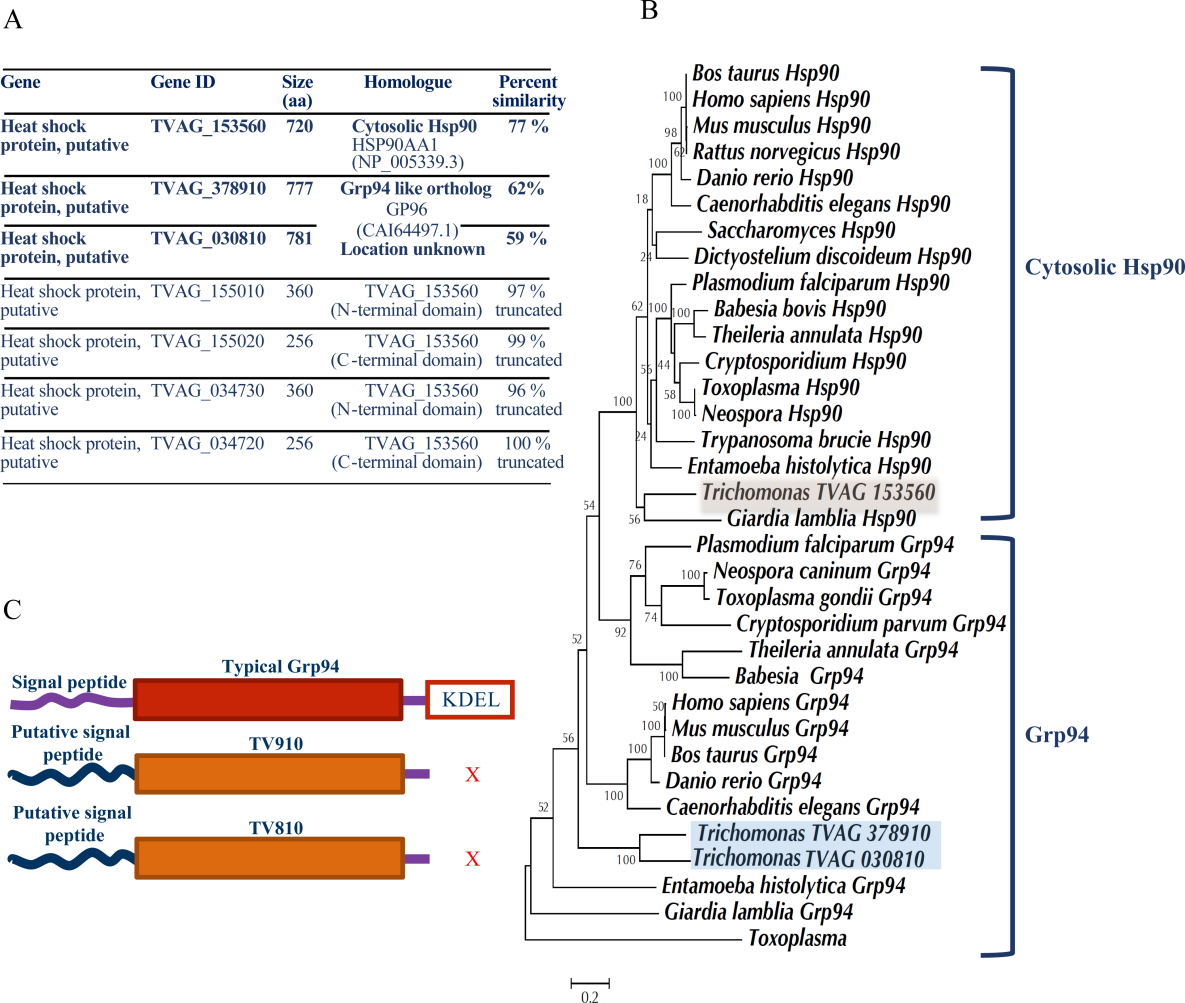
Hsp90 isoforms in *Trichomonas vaginalis*. (A). The table shows gene IDs and features of seven Hsp90 isoforms identified in *T*. *vaginalis*. There is one full-length cytosolic Hsp90, four truncated homologs of cytosolic Hsp90 and two isoforms that show homology to Grp94 but lack an ER retention signal. The table also shows percent similarity of each of the genes to the homologs indicated in the table (B). TVAG_378910 and TVAG_030810 are Grp94 orthologs. The phylogenetic tree was constructed using maximum likelihood algorithm using Hsp90 and Grp94 sequences from different organisms. TVAG_153560 clusters with cytosolic Hsp90s and TVAG_378910 and TVAG_030810 clusters with Grp94s. The percentage of trees in which the associated taxa clustered together is shown next to the branches. (C).TV910 and TV810 lack an ER retention signal. TV560 has conserved DDVD motif at the C-terminus. However, TV910 and TV810 both lack an ER retention signal (see S1 Fig for the alignment).

There are seven genes annotated as Hsp90 in TrichDB.org (Fig 1A). Amongst the 7 annotated genes, 5 genes are homologous to cytosolic Hsp90. Among these 5 cytosolic isoforms, there is only one gene, TVAG_153560 (TV560), which codes for a full-length Hsp90 with 721 amino acids and is an intron less ORF of 2.1 kb. The other 4 genes are the products of non-sense mutation leading to premature termination of Hsp90, which if expressed can code for truncated Hsp90 with an N-terminal domain and a part of the middle domain. These nonsense mutations in genes TVAG_155010 and TVAG_034730 have resulted in the C-terminal halves to being annotated as separate genes TVAG_155020 and TVAG_034730.

Two of the remaining genes, TVAG_030810 and TVAG_378910 showed significant identity to Grp94, an ER resident paralog of Hsp90. Both the genes were present on two different scaffolds, DS113541 (TVAG_030810) and DS113184 (TVAG_378910). To ascertain their identity, phylogenetic analysis was carried out. A phylogenetic tree was constructed using the sequences of well-annotated cytosolic Hsp90s and Grp94 using the maximum likelihood method. These sequences were retrieved by BLASTp analysis using human cytosolic Hsp90 and Grp94 as queries in NCBI and EupathDB databases. The tree shows that full-length cytosolic Hsp90 of *Trichomonas vaginalis* (TVAG_153560) clustered with cytosolic Hsp90s. However, both TVAG_030810 and TVAG_378910 clustered with Grp94 clade (Fig 1B). Any potential homology with TRAP1 (a mitochondrial Hsp90 paralog) was also ruled out by sequence and phylogenetic analysis (S2 Fig).

### Grp94 homologs, TV910 and TV810, lack the canonical ER retention signal

For our further analysis, we focused only on the three full-length Hsp90 isoforms, cytosolic Hsp90 TV560, and Grp94 orthologs, TV910 and TV810. We performed sequence analysis and aligned the three isoforms using MUSCLE algorithm. Cytosolic Hsp90 had a canonical DDVD motif at the C-terminal end, which is conserved and is responsible for binding to co-chaperones and clients containing TPR domain. However, upon a closer look, we noticed that both the Grp94 orthologs TV910 and TV810 showed neither a canonical ER signal peptide at the N–terminus, nor a retention sequence at the C–terminus of the protein (Figs 1C and S1). It has been noticed in *Trichomonas* that many of the ER and exported proteins lack a canonical signal sequence [23, 24]. In the alignment (S1 Fig), we noticed TV810 and TV910 have additional 46 and 18 residues at the start of their N-termini, respectively. These extra nucleotides at the start of the N-terminal domain encoded by these genes could possibly indicate signal peptide (Fig 1C).

ER resident proteins are characterized by the presence of an ER retention signal KDEL (or sequence variants of this signal). However, both TV810 and TV910 lack this ER retention sequence (Figs 1C and S1). When we analyzed sequences of other conserved ER resident proteins of *Trichomonas* like Bip (TVAG_092490), calreticulin (TVAG_122020) *etc*. we found them to have an ER retention signal. Overall, we could count a total of 171 proteins in *Trichomonas vaginalis* to have an ER retention signal KDEL or variants (sequences ending with DEL, EEL, EDL, and DDL) (S1 Table). We also searched for the homolog of KDELR1 (KDEL endoplasmic reticulum protein retention receptor 1) in the *Trichomonas* genome. This receptor protein recognizes the ER retention signal and retrieves resident soluble proteins from the Golgi [25]. We could identify a homolog of KDELR1, TVAG_242900, in the *Trichomonas* genome. These observations suggested that the sorting mechanism of ER resident proteins is putatively conserved in *Trichomonas*. Overall, our observations suggest that Grp94 orthologs in *Trichomonas* lack an ER retention signal and do not share this peculiarity with other bonafide ER resident proteins of *Trichomonas*.

To the best of our knowledge no other study describes a Grp94 ortholog lacking an ER retention signal. To check if the absence of an ER retention signal is a rare occurrence limited to *Trichomonas* Grp94s, or is a more common phenomenon we analyzed Grp94 homologs from various taxonomic clades of Protista including *Alveolata*, *Amoebozoa*, *Apicomplexa*, *Ciliophora*, *Diplomonadida*, *Euglenozoa*, *Myxospora*, *Parabasalia* and related *microsporidia*. Human Grp94 and TV910 were used as the query for BLASTp search in NCBI database for above-mentioned taxa. A total of 913 sequences were retrieved. From these putative Grp94 sequences, all proteins containing ER retention signal and its variants at the C-terminus (DEL, EEL, EDL & DDL) were eliminated. A second round of filtering was carried out for cytosolic Hsp90s, mitochondrial Trap1, or apicoplast Hsp90 (the organellar Hsp90 paralog of apicoplast), showing high similarity to Grp94, by reverse BLAST analysis. Final validation of an ER retention signal lacking Grp94s was done by constructing a phylogenetic tree using sequences of annotated Hsp90 paralogs of different cellular organelles and cytosol using the maximum likelihood method.

The analysis revealed that only 5 sequences of Grp94 lacked an ER retention signal among those analyzed (S2 Fig). These sequences belong to three genera. Two of them were TV910 and TV810 from *Trichomonas vaginalis*, two from *Mitosporidium daphniae* (KGG51005.1 and KGG52886.1) and one from *Ichthyophthirius multifiliis* (MG5_078890). This shows that the absence of an ER retention signal in Grp94 is indeed a rare phenomenon and warranted further investigation. The absence of an ER retention signal further suggests that these particular Grp94 orthologs in *Trichomonas* may not be retained in ER.

### *Trichomonas* expresses both Grp94 orthologs

**Fig 2:**
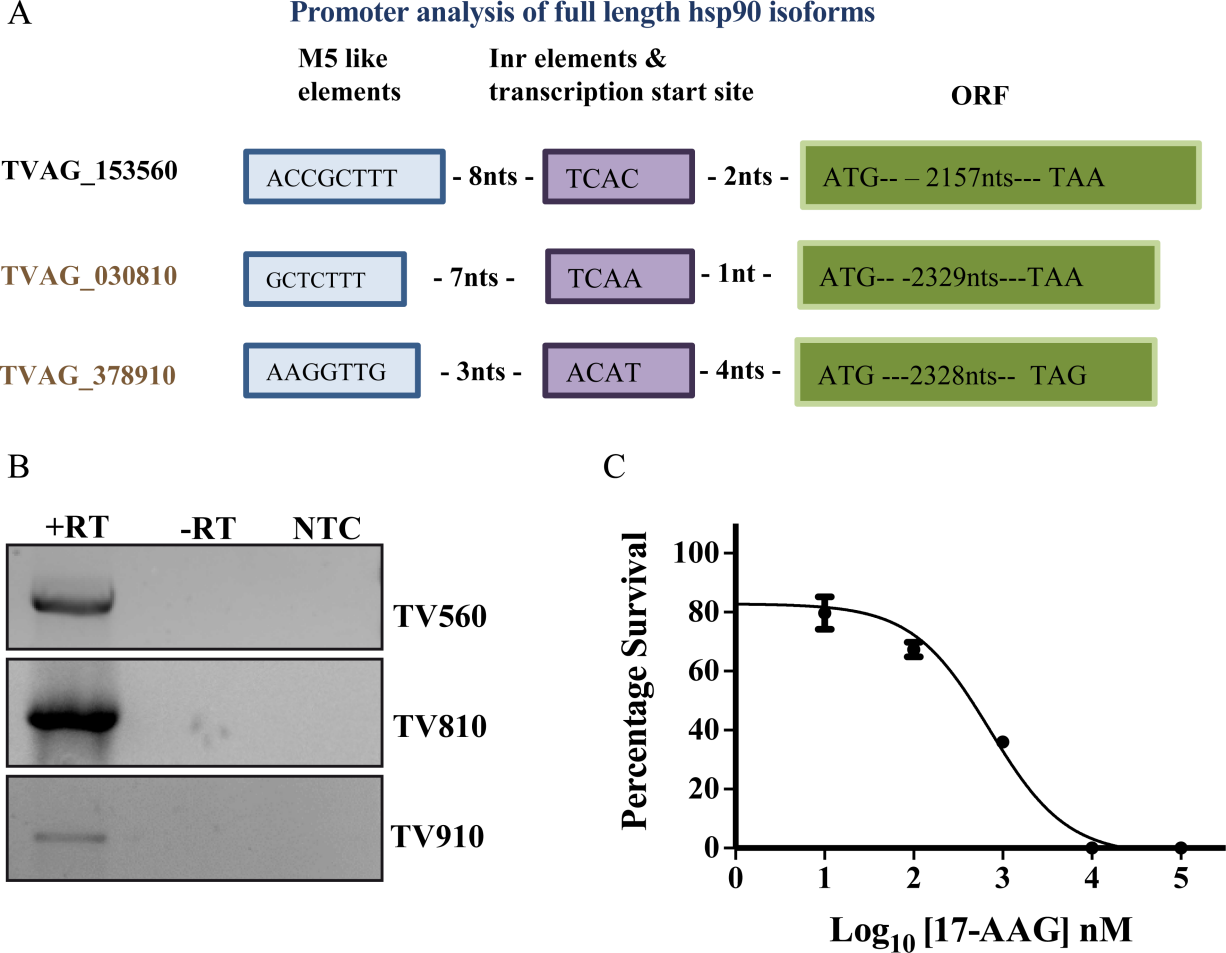
*Trichomonas* expresses all three full-length Hsp90 isoforms and depends on Hsp90 for its growth. (A) Sequence analysis upstream of the three full-length Hsp90 isoforms in *T*. *vaginalis* shows the presence of conserved M5-like and Inr elements. (B) Agarose gel shows RT- PCR amplicon for full-length transcripts of TV560, TV810, and TV910, thus confirming their expression at the transcript level. Lane-1: PCR using respective primers from cDNA, Lane 2: no Reverse transcription control to control for genomic DNA contamination, Lane 3: no template control. (C) GI_50_ for growth arrest by 17-AAG was determined by counting viable cells after 24 h treatment with the drug. The graph shows growth inhibition of *Trichomonas* by 17-AAG for with a GI_50_ of 708 nM.

Pseudogenes are common in the vastly expanded genome of *Trichomonas*, most of these pseudogenes are due to absence of promoter elements. In comparison with the promoter elements identified in *Trichomonas*, both the Grp94 orthologs, TV810 and TV910, along with cytosolic Hsp90 TV560, were found to be under the control of similar promoter-like elements (Fig 2A). Sequences similar to M5-like elements and Inr elements were found to be present upstream of all the three Hsp90 isoforms [26].

Further, expression of all three isoforms was confirmed by RT PCR. Briefly, total RNA of *Trichomonas* trophozoites was isolated and cDNA was prepared. The presence of TV560, TV810 and TV910 mRNA was confirmed by gene-specific primers by RT PCR. Fig 2B shows PCR amplicons of full-length genes for all three isoforms. This confirms that both the Grp94 orthologs which lack an ER retention signal, in addition to cytosolic Hsp90, are expressed by *T*. *vaginalis* and are not pseudogenes.

### Hsp90 isoforms in *Trichomonas* are functional ATPases

We further investigated if a functional Hsp90 is required for parasite growth. Therefore, to examine the effect of Hsp90 inhibition on growth, *Trichomonas* trophozoites were grown to log phase then treated with varying concentrations of 17-AAG in the range of 10 nM to 100 µM for 24 hours. Cell survival was measured by counting viable cells using trypan blue dye exclusion methodology. Percent survival was plotted against Log_10_ [17-AAG] concentration. Complete cell death was observed at higher drug concentrations. Growth inhibitory concentration GI_50_ for 17-AAG treatment was 708 nM (Fig 2C). This shows a functionally important role for Hsp90 in *Trichomonas*.

**Fig 3:**
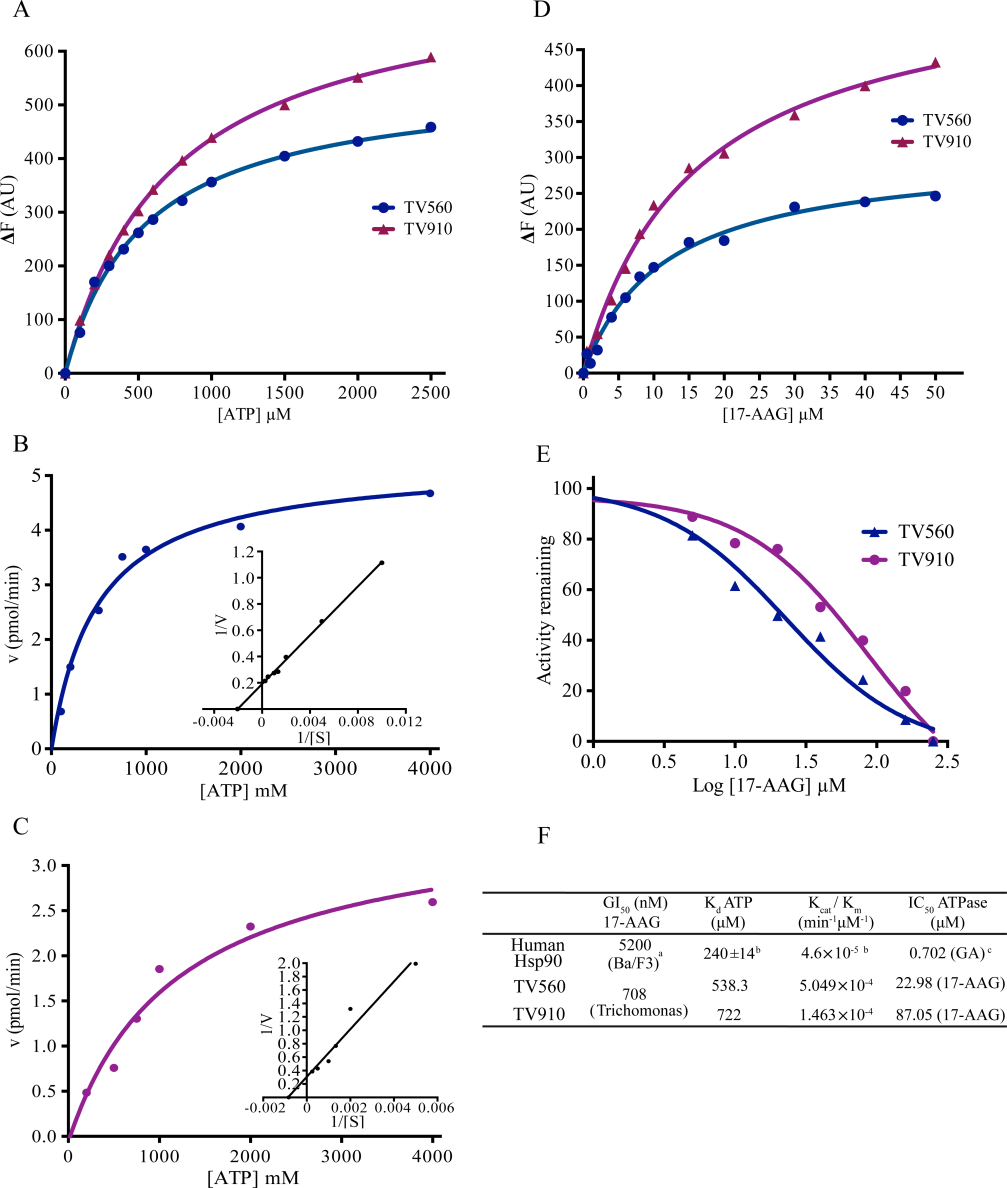
Both cytosolic Hsp90 TV560 and the Grp94 isoform TV910 are functional ATPase *in vitro*. (A) The binding affinity of the natural ligand ATP to Hsp90s was determined using tryptophan fluorescence quenching. Changes in intrinsic fluorescence intensity upon ligand binding was plotted against ligand concentration. The dissociation constant, k_d_, for ATP binding, was found to be 538.3 μM for TV560 and 722 μM for TV910. (B) & (C) The rate of ATP hydrolysis was measured by the hydrolysis of ATP to ADP where γ^32^P- labeled ATP was used as a tracer. A Michaelis–Menten plot shows the rate of ATP hydrolysis plotted against ATP concentration. Inset graph is the representative Lineweaver–Burke plot for each of the Hsp90’s ATPase activities. (B) ATPase activity of TV560 and (C) ATPase activity of TV910. (D) The binding affinities of pharmacological inhibitor 17-AAG to Hsp90s were determined using tryptophan fluorescence quenching. The dissociation constant, k_d_, for 17-AAG binding, was found to be 11.45 μM for TV560 and 15.73 μM for TV910. (E) 17-AAG potently inhibits ATPase activity of cytosolic parasitic Hsp90s. ATPase assay was performed in the presence of increasing concentrations of 17-AAG and percent remaining activity was calculated. Graphs show TV560 inhibition by 17-AAG with an IC_50_ of 22.98 μM, and TV910 inhibition by 17-AAG with an IC_50_ of 87.05 μM. (F) The table shows a comparison of biochemical parameters of TV910 and TV560 with human Hsp90 ^a^[27], ^b^[28], ^c^[8].

Grp94 orthologs TV810 and TV910 show high conservation and both lack an ER retention signal and possess a putative signal sequence (Fig 1C), therefore, for all further experiments, only one isoform was investigated, and we chose TV910 for this purpose and compared its biochemical parameters with cytosolic Hsp90 TV560. We showed that both TV560 and TV910 can bind ATP with a strong affinity with a k_d_ of 538.3 µM and 722 μM, respectively (Fig 3A). We then measured the catalytic activity of the two Hsp90 isoforms. Briefly, ATPase activity of Hsp90s was analyzed by incubating purified Hsp90s with increasing concentrations of ATP. γ^32^ P-ATP was used as a tracer, and ATP hydrolysis rate was analyzed on a TLC plate. Fractional cleavage of ATP by Hsp90 was used to calculate enzyme velocity, which was plotted against corresponding ATP concentrations. In the control reactions, 300 μM of the Hsp90 inhibitor, 17-AAG, was added to inhibit Hsp90 activity and reveal only background activity, which is subsequently subtracted from the activity of Hsp90 to negate nonspecific background. The data was analyzed by GraphPad Prism using non-linear regression analysis for Michaelis-Menten kinetics (Fig 3B and 3C). K_M_ for TV560 and TV910 were found to be 486.6 µM and 1206 µM, respectively. TV910 showed a higher K_M_ value compared to TV560. The k_cat_ was found to be similar at 0.263 min^-1^ and 0.176 min^-1^ respectively for TV560 and TV910. The catalytic efficiency was found to be 5.049×10^−4^ min^-1^µM^-1^ and 1.463 ×10^−4^ min^-1^µM^-1^ respectively for TV560 and TV910 suggesting cytosolic Hsp90 is a more active ATPase. 17-AAG is a known inhibitor of Hsp90 ATPase. The dissociation constant (k_d_) for 17-AAG was observed to be 11.45 µM and 15.73 µM respectively for TV560 and TV910 (Fig 3D). 17-AAG could inhibit the ATPase activity of both the Hsp90 isoforms, though weakly for TV910 (IC_50_: 87.05 µM) when compared to cytosolic Hsp90 (IC_50_: 22.98 µM). (Fig 3E). Overall, our biochemical analysis suggests that both cytosolic Hsp90 and the Grp94 ortholog TV910 are active *in vitro*. Compared to human Hsp90, *Trichomonas* Hsp90s are more active ATPases (Fig 3F).

### *Trichomonas* secretes TV910

Our observations suggest that TV910 encodes an Hsp90 isoform with functional ATPase activity. To study the localization of TV910 in *Trichomonas*, an antibody against full-length recombinant TV910 was raised in rabbit. The anti-TV910 antibody was purified from rabbit serum using Protein-A affinity column. Antibody α-TV910 was found to be specific to TV910 and did not cross-react with purified TV560 or TV810 proteins (S3 Fig).

A hallmark of secreted proteins is the presence of a signal sequence which is recognized by SRP (signal recognition particle). The newly synthesized polypeptide is translocated to ER with the aid of SRP and SRP receptor on the ER membrane. The protein is folded in the ER and is transported to Golgi apparatus by COPII coated vesicles and those secretory proteins lacking an ER retention signal are further secreted through Golgi [29]. As described before, TV910 is a Grp94 homolog with an extended N-terminal sequence that could potentially be a signal peptide and lacks an ER retention signal (Figs 1C and S1), therefore, we hypothesized that probably TV910 is secreted by *Trichomonas*.

**Fig 4:**
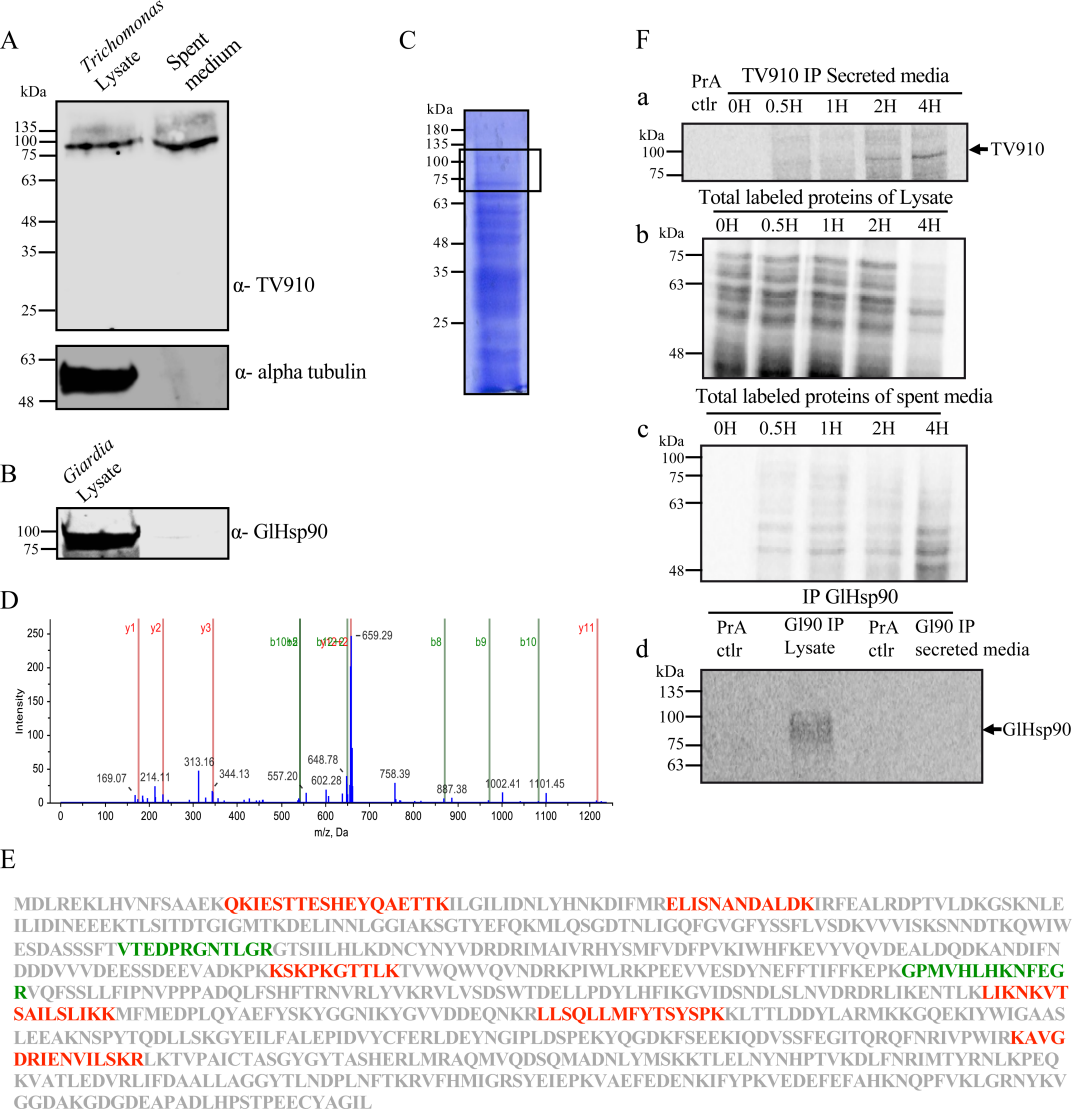
TV910 is secreted by *Trichomonas*. **(**A) Immunoblot shows TV910 in the cellular lysate and spent medium consistent with the secretion of TV910. No signal for alpha-tubulin was observed in spent media confirming cellular integrity. (B) Immunoblot for lysate and spent medium of *Giardia* trophozoites shows a prominent signal in the lysate and no signal in spent medium. (C) Coomassie profile of total secretome of *Trichomonas vaginalis*. (D) MS/MS spectra of the peptide ‘VTEDPRGNTLGR’ of TV910 detected in the tryptic digest corresponding to 75–100 kDa region. (E) TV910 sequence showing the peptides identified by MS/MS analysis. Peptides in green were identified with a confidence of 95% or more. (F) TV910 is secreted in a time-dependent manner. (a) Autoradiograph of IP of TV910 in the spent medium for different time points following the 2 H pulse of labeling. Protein A lane does not show any signal. A time-dependent increase is seen in the TV910 signal. (b) Autoradiograph of total labeled proteins of lysate shows a time-dependent decrease. (c) Autoradiograph of total labeled secreted proteins shows a time-dependent increase in signal. (d) Autoradiograph of IP for GlHsp90 after pulse-chase shows signal only in the lysate and no signal in spent media.

To check if TV910 is indeed secreted, *T*. *vaginalis* trophozoites were harvested at log phase of growth. They were washed thrice with PBS to remove any medium and resuspended in PBS-sucrose (5% w/v) and incubated for 3 hours at 37°C at a cell density of 10^5^ cells/ml. Cells were pelleted and lysed and spent media was filtered using 0.22 μM filter and concentrated using Amicon ultra-15 filters. Both lysate and spent media were resolved on an SDS-PAGE followed by western transfer. The blot was probed with the α-TV910 antibody. A clear signal for TV910 was observed at its molecular weight of ~89.5 kDa in both the lysate and spent medium (Fig 4A). As a control to rule out any lysis and leakage of cellular content into the spent media, the blot was probed with α-alpha tubulin for which the signal was observed only in the lysate and not in the spent media (Fig 4A). Alpha tubulin has been previously shown not to be present in spent media of *T*. *vaginalis* and is used as a control for cellular integrity [23, 30]. As an additional control, a secretion assay was set up for *Giardia lamblia* trophozoites. *Giardia* is an early branching close relative of *Trichomonas*. The experiment was set up in the same way as described for *Trichomonas*. A very prominent signal for GlHsp90 was observed in the cell lysate, but not in spent media (Fig 4B).

To further validate secretion of TV910, proteins present in spent media were resolved on an SDS-PAGE and stained with Coomassie brilliant blue (Fig 4C). The gel region between 75–100 kDa was excised and trypsin digestion was performed. The tryptic digest was analyzed on an LC-MS/MS ESI- QTOF system. Data was analyzed using Protein Pilot 5 against *Trichomonas* database downloaded from NCBI. TV910 was identified in spent media with high confidence. Fig 4D shows MS/MS spectrum of peptide “VTEDPRGNTLGR” that was identified with a confidence of more than 95%. Fig 4E shows the TV910 sequence and peptides identified highlighted in color. Thus, we confirmed by MS/MS that TV910 is indeed secreted by *Trichomonas* into the spent medium.

We also developed an MRM-based method to detect TV910 in the secreted medium. The tryptic digest from blank (only PBSS) and the spent medium were analyzed using Agilent 1260 Infinity LC coupled with 6460 Triple Quad MS. Based on the detection of the two unique peptides, our analysis showed the presence of TV910 in the spent medium; further strengthening our biochemical data (S4 and S5 Figs). Analysis using MRM can be further used to quantitate levels of protein(s) in the spent medium and can be a robust alternative method to antibody-based detection.

Steady-state levels show the presence of TV910 in the secreted medium. To further study the kinetics of secretion, a pulse-chase experiment was performed. Briefly, cells were starved in cysteine- and serum-free medium for 2 hours following which they were pulsed for two hours by metabolic labeling of proteins using S^35^ containing Met-Cys mix. Following the pulse, cells were washed and labeled medium was removed. Labeled cells were divided into five equal aliquots and a chase in PBS-sucrose (5% w/v) was carried out at 37°C for different time points (0 H, 0.5 H, 1 H, 2 H and 4 H). Labeled TV910 was pulled down from spent media using α-TV910 antibody. Signal was detected using autoradiography.

Fig 4F (a) shows the immunoprecipitation profile of TV910 at different time intervals. It can be clearly seen that signal for TV910 starts to appear 2 H and increases by 4 H. At 0 H no signal was observed for TV910. At 0.5 H and 1 H also there is no significant signal for TV910. Fig 4F (b) shows the total profile of labeled proteins in the cell. A decrease in the labeled proteins can be seen by increasing time. Total protein content was equal in all the lanes. Fig 4F (c) shows the total profile of labeled proteins in spent media at different time points of the chase in the unlabeled medium. A clear increase in total secreted labeled proteins can be seen by increasing time. As an additional control, pulse-chase (pulse of 2 H and chase of 4 H) was performed for *Giardia* trophozoites, as described above followed by IP for GlHsp90. No signal for GlHsp90 was seen in spent media and GlHsp90 was pulled down only in case of lysate (Fig 4F d). Overall, we show that TV910 is actively secreted by *Trichomonas* in a time-dependent manner.

### TV910 is secreted via the classical secretory pathway

**Fig 5:**
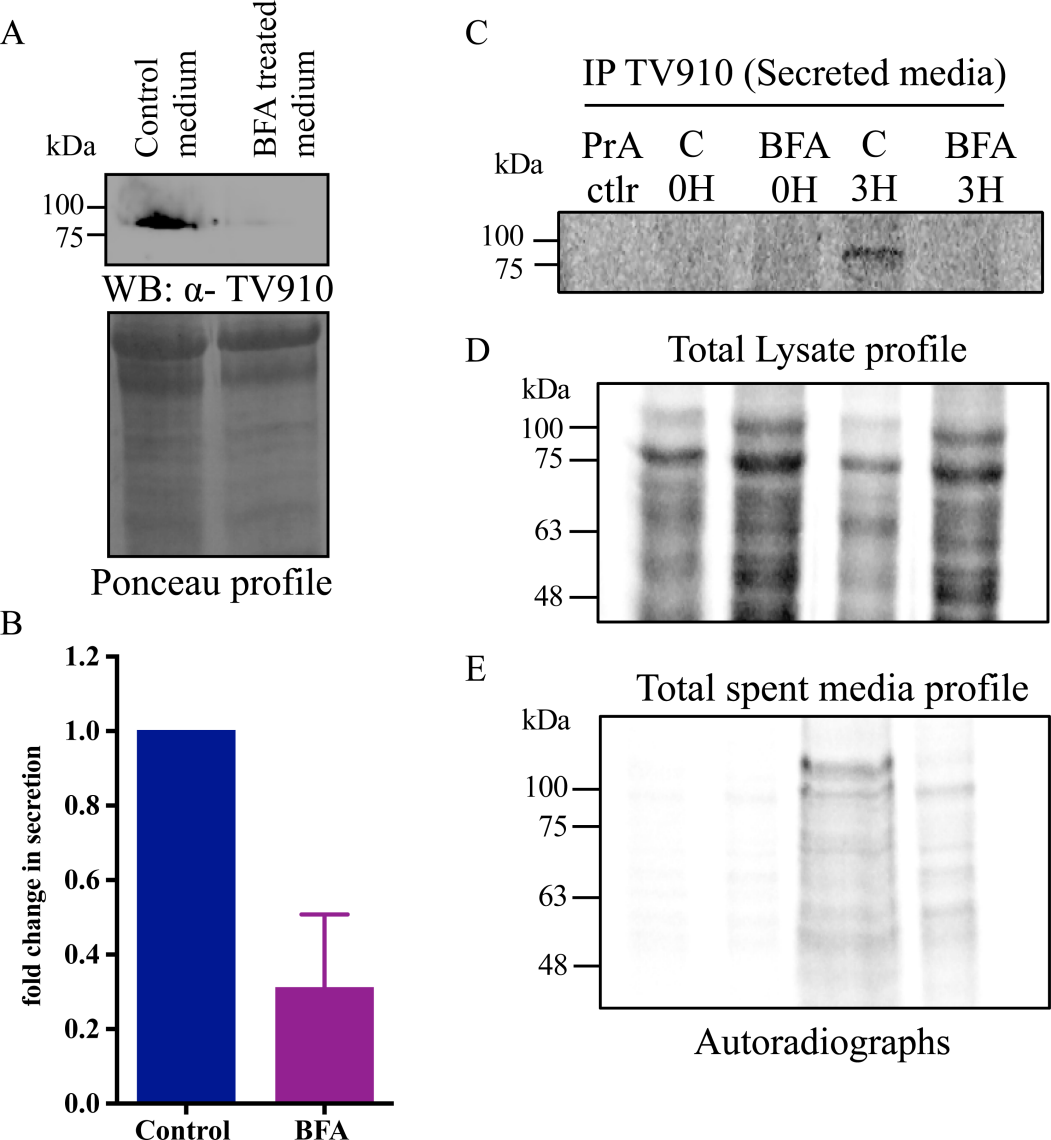
TV910 is secreted via a BFA-sensitive secretory pathway. (A) Immunoblot shows inhibition of TV910 secretion into medium upon BFA treatment. Ponceau profile is shown as loading control. (B) The graph shows quantitation of signal. (C) Autoradiograph of IP for secreted TV910 at 0 and 3 H of the chase after 2 H pulse of S^35^ labeling with and without BFA treatment. Upon BFA treatment no signal for secreted TV910 was observed at 3 H. (D) Autoradiograph of total labeled proteins of lysate shows a time-dependent decrease from 0 to 3 H. Upon BFA treatment higher signal was observed in cells due to inhibition of signal. (E) Autoradiograph of total labeled secreted proteins show a time-dependent increase in signal and upon BFA treatment a clear decrease was seen in signal due to inhibition of secretion.

To study if TV910 is secreted via a classical ER–Golgi secretory pathway, secretion assays were performed in the presence of Brefeldin-A (BFA). BFA is a fungal metabolite that blocks the formation of COP-I coated vesicles by inhibiting the small GTPase Arf1p. This causes redistribution of Golgi proteins and thus, inhibits classical ER-Golgi pathway [31, 32]. We looked at the effect of BFA on TV910 secretion using two different approaches. Firstly, the effect of BFA was tested on steady state levels of TV910 immunoblot. Cells were treated with 10 μg/mL of BFA for 2 hours, followed by secretion assay in PBS-sucrose for 3 hours as described before. An equal number of cells were treated with vehicle control (ethanol). Spent media was filtered and concentrated. Total secreted proteins for control and BFA-treated cells in spent media were resolved on SDS PAGE followed by western blot. Fig 5A and 5B show blot for TV910 in control treated spent media and BFA-treated spent media and quantitation of TV910 signal, respectively. A clear decrease can be seen in TV910 secretion upon BFA treatment. Ponceau profile shows equal loading.

Further, a labeled pulse-chase experiment was performed for TV910, as described in the previous section with a pulse of two hours and chase of 0 and 3 hours. Cells were treated with vehicle (ethanol) or BFA for two hours during the pulse with S^35^-Met-cys label. This was followed by an IP for TV910. As can be clearly seen in Fig 5C, no signal for TV910 was seen at 0 H in both control and BFA-treated spent media, however, at 3 H there is a clear signal for TV910 in control and no signal in BFA-treated spent media. Fig 5D shows total labeled proteins in the lysate and there is a decrease in the amount of labeled proteins from 0 H to 3 H as expected. There is a comparatively higher amount of labeled proteins in BFA-treated cells, which is due to inhibition of secretion and retention of more proteins in the cell compared to control. Fig 5E shows that there is an increase in the amount of total labeled proteins from 0 to 3 H in spent media of control cells, and at 3 H, spent media of BFA-treated cells have a lesser amount of labeled protein as expected. These results altogether suggest that BFA blocks TV910 secretion. Thus, we can conclude that TV910 follows classical ER-Golgi pathway for secretion.

## Discussion

*Trichomonas vaginalis* is a clinically important parasite and a common cause of STD, with millions of new infections worldwide every year. The problem of STD is compounded by poor hygiene in lower socio-economic classes, lack of good healthcare facility and social taboo associated with it. These limitations prevent the timely treatment of disease in many patients. *Trichomonas* ranks highest in terms of prevalence and incidence among the four major non-viral treatable sexually transmitted infections that include *Chlamydia trachomatis*, *Neisseria gonorrhoeae*, and *Treponema pallidum*.

*Trichomonas vaginalis* has to deal with constant stress in its physiological niche of the urogenital tract that includes changes in pH, fluctuation in iron balance and other nutrients and desquamation of vaginal epithelial cells associated with menstrual cycle. In this study, we show that the parasite critically depends on Hsp90 for its growth and Hsp90 inhibition is lethal. We identified Grp94 orthologs lacking an ER retention signal. We show that TV910 is actively secreted by the parasite into the extracellular milieu. We provide biochemical and proteomics evidence for the secretion of this Hsp90 isoform. We observed that lack of an ER retention signal in a Grp94 ortholog is a rare phenomenon. Other canonical ER resident proteins of *Trichomonas* possess the conserved ER retention signal at their C-termini and the ER sorting machinery appears to be conserved at least at the genomic level. We show that TV910 follows the classical ER-Golgi secretory pathway and inhibition of this pathway by BFA blocks its secretion. To our knowledge, this is the first genome-annotated Hsp90 to be secreted by a parasitic protozoan.

We analyzed mass-spectrometry data from our studies and other reports [23, 33] for other secreted proteins of *Trichomonas* and found potential Hsp90 interactors in the secretome, including Hsp70, peptidyl- prolyl-isomerase that are the known co-chaperones of Hsp90. It will be of interest to see if the extracellular TV910 isoform can interact with them. Many potential Hsp90 client proteins, including metabolic and signaling molecules, are also present in the secreted proteome [23, 33].

For establishing infection, *Trichomonas* requires to break through the mucus layer and adhere to epithelial cells of urogenital tract [34]. It also penetrates into basement membrane and binds to extracellular matrix proteins [35]. The role of extracellular Hsp90 has been previously shown to be important in migration and invasion of tumor cells [16, 17, 20]. Grp94 has also been shown to have a tumor-specific cell surface expression [15, 36]. Inhibition of extracellular Hsp90 using cell-impermeable Hsp90 inhibitor FITC- Geldanamycin [21] has no apparent effect on the growth of the parasite in the axenic culture conditions (S6 Fig). This is not surprising as one would expect the intracellular Hsp90 to be essential for growth which we have shown in this study. However, based on the functions described for Hsp90 isoforms in the extracellular space, we can speculate that extracellular Hsp90 may play an important role in virulence and pathogenesis of *Trichomonas* while infecting the host and this needs to be investigated.

One of the major reasons for the high prevalence of *Trichomonas* infection is the lack of proper diagnosis of the infection due to various reasons including absence of reliable diagnostic methods, especially in developing countries. Based on communication with many clinicians, we found out that there is an absence of a defined diagnostic strategy, and often diagnosis happens based on symptoms. Very few labs carry out microscopic observations for parasites in infected samples. One of the major implications for secreted Hsp90 of *Trichomonas* is the possibility of its use as a biomarker to detect infection and our MRM based detection method can be further tested in clinical settings.

Overall, we show that *Trichomonas* Grp94 orthologs lack the ER retention signal and the parasite secretes Hsp90. To date, major focus on extracellular Hsp90 has been in the context of cancer. We show that a protozoan parasite is actively secreting Hsp90 which may have an important role in its virulence and survival.

## Supporting information

S1 Fig Sequence alignment of Hsp90 isoforms of *Trichomonas*. (JPG)

S2 Fig Absence of ER retention signal in Grp94 is not common. Sequence and phylogenetic analysis show only 5 Grp94 sequences from a total of 913 analyzed sequences lacked ER retention signal. Three of these sequences are highlighted in the zoom out of the phylogenetic tree. The alignment shows the C-terminus of Grp94s and highlighted Grp94 sequences from *Mitosporidium*, *Ichthyophthirius* and *Trichomonas* were among five Grp94s identified which lacked ER retention signal.
(JPG)

S3 Fig Blot showing specificity of the α-TV910 antibody. (JPG)

S4 Fig Total ion chromatograms for TV910 peptide DELINNLGGIAK. (a) TIC of pure TV910 indicates the MRM 628–9 > 786.5 for the peptide DELINNLGGIAK and the RT of 18.116 min. (b) TIV of blank (only PBSS) does not show any high-intensity peak at the specific MRM and RT. (c) TIC of the spent medium at the specific MRM and RT is indicative of the presence of the peptide of TV910 in the secreted medium of *Trichomonas*.
(TIF)

S5 Fig Total ion chromatograms for TV910 for peptide IENVILSK. (a) TIC of pure TV910 indicates the MRM 458.3 > 673.4 for the peptide IENVILSK and the RT of 17.281 min. (b) TIV of blank (only PBSS) does not show any high-intensity peak at the specific MRM and RT. (c) TIC of the spent medium at the specific MRM and RT is indicative of the presence of the peptide of TV910 in the secreted medium of *Trichomonas*.
(TIF)

S6 Fig *T*. *vaginalis* cells were treated with FITC-GA at various concentrations. Post 24- and 36-hrs, cell survival, and viability were measured. Inhibition of extracellular Hsp90 did not significantly compromise cell survival and viability.
(TIFF)

S1 Table Analysis of *Trichomonas vaginalis* proteins which contain an ER retention motif at their C-terminus. (DOCX)

S2 Table The table summarizes the conditions used for Multiple Reaction Monitoring (MRM) for the two peptides (DELINNLGGIAK and IENVILSK) of TV910 of *Trichomonas vaginalis*. (DOCX)

S3 Table The table summarizes the chromatographic conditions used for the detection of two peptides using the MRM method. (DOCX)
