# Supplementary figures and images for: A secreted Heat shock protein 90 of Trichomonas vaginalis
Source: PLoS Negl Trop Dis. 2018 May 16;12(5):e0006493. doi: 10.1371/journal.pntd.0006493 (PMC5973626; doi:10.1371/journal.pntd.0006493)

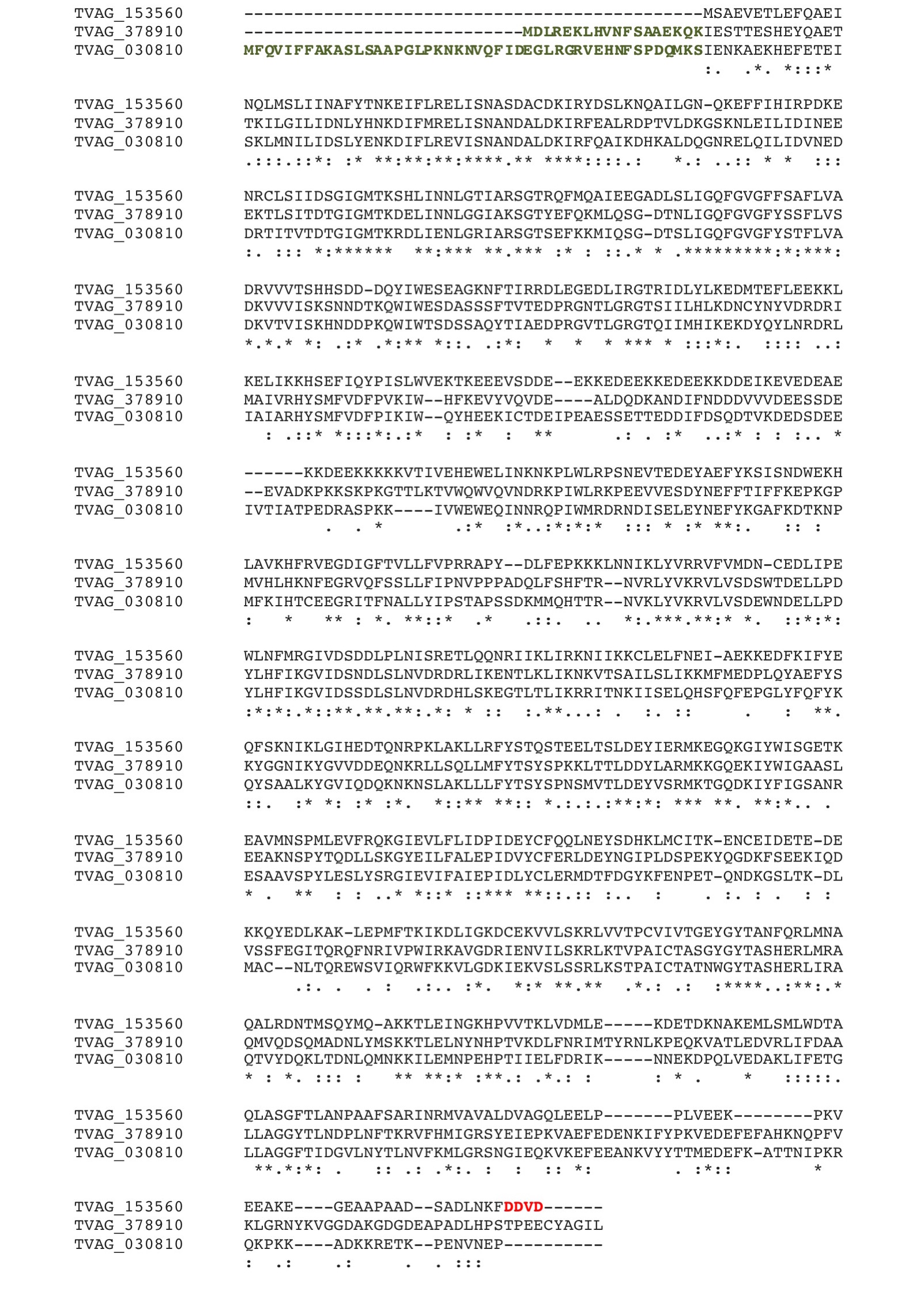

Supplement: S1 Fig — (JPG) [file pntd.0006493.s001.jpg]

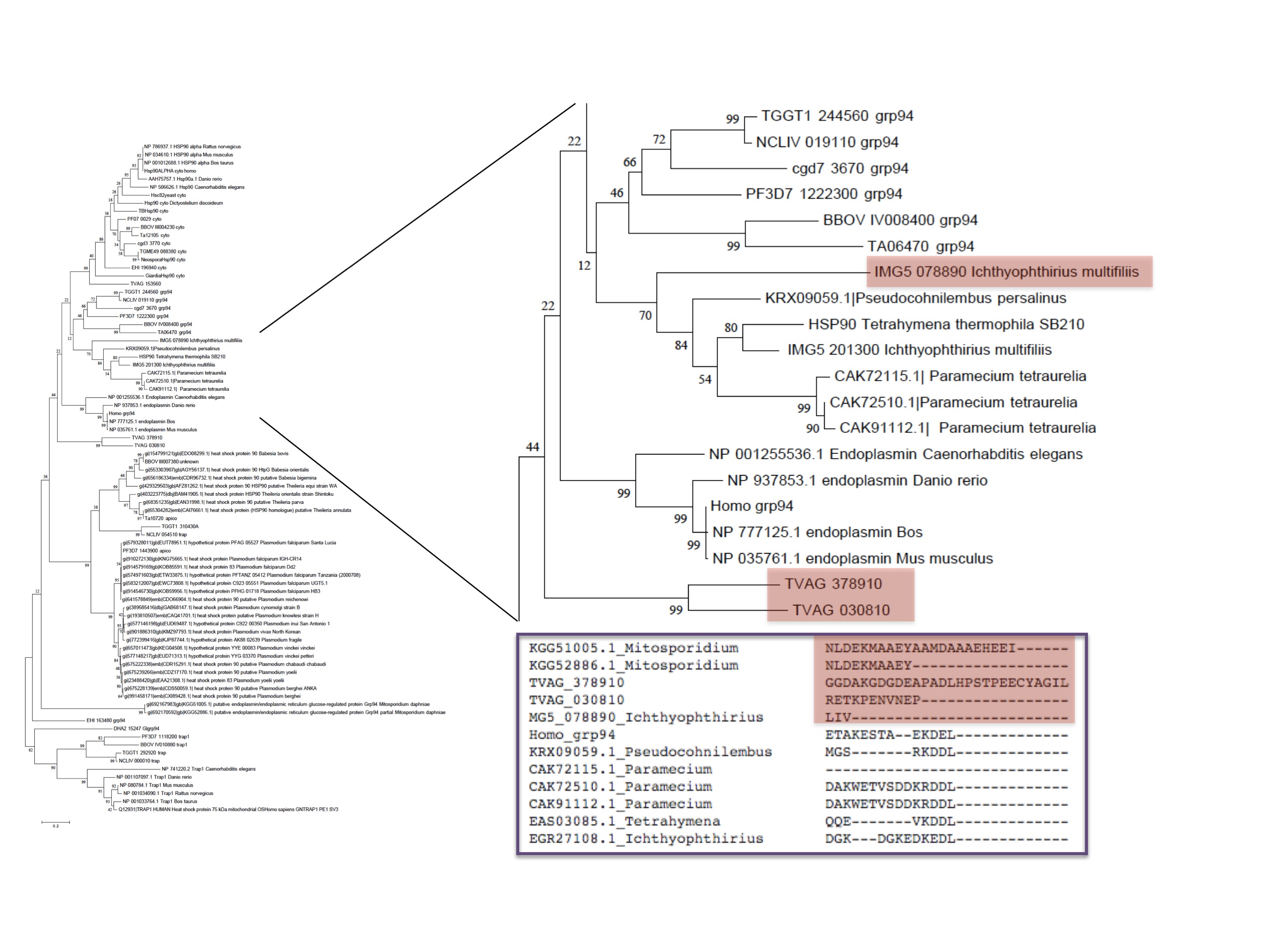

Supplement: S2 Fig — Sequence and phylogenetic analysis show only 5 Grp94 sequences from a total of 913 analyzed sequences lacked ER retention signal. Three of these sequences are highlighted in the zoom out of the phylogenetic tree. The alignment shows the C-terminus of Grp94s and highlighted Grp94 sequences from Mitosporidium, Ichthyophthirius and Trichomonas were among five Grp94s identified which lacked ER retention signal. (JPG) [file pntd.0006493.s002.jpg]

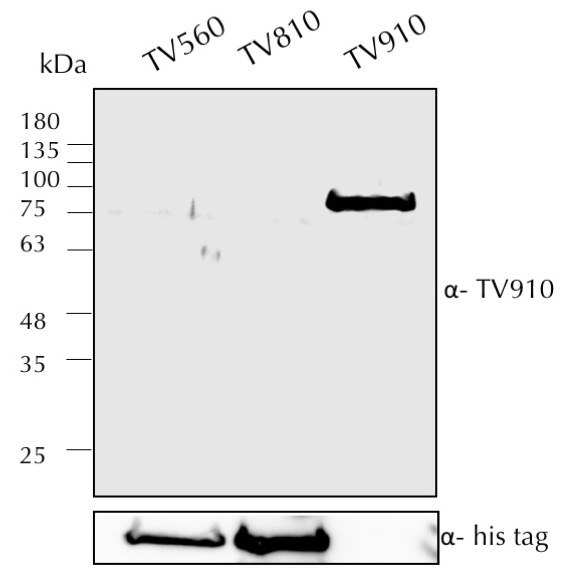

Supplement: S3 Fig — (JPG) [file pntd.0006493.s003.jpg]

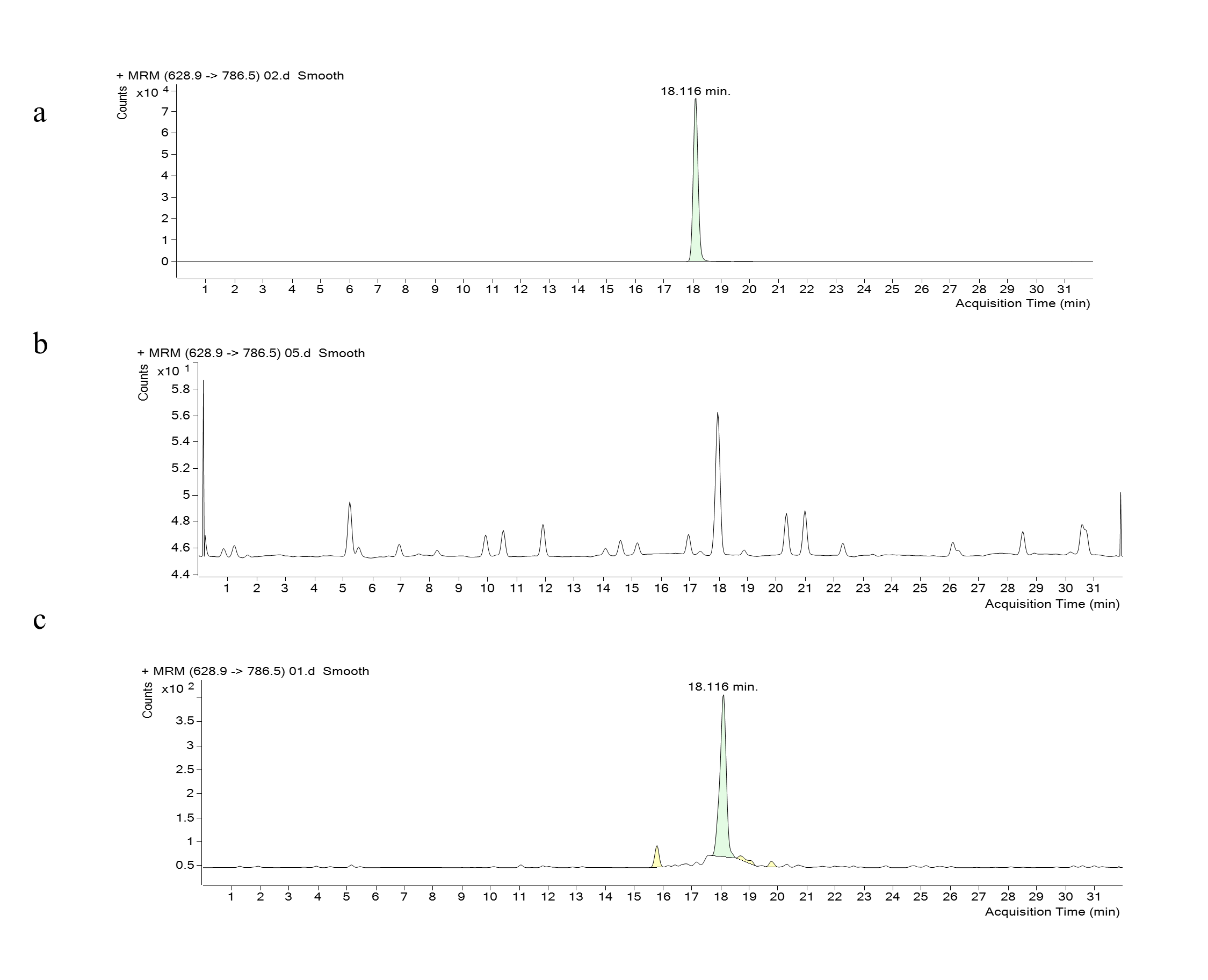

Supplement: S4 Fig — (a) TIC of pure TV910 indicates the MRM 628–9 > 786.5 for the peptide DELINNLGGIAK and the RT of 18.116 min. (b) TIV of blank (only PBSS) does not show any high-intensity peak at the specific MRM and RT. (c) TIC of the spent medium at the specific MRM and RT is indicative of the presence of the peptide of TV910 in the secreted medium of Trichomonas. (TIF) [file pntd.0006493.s004.tif]

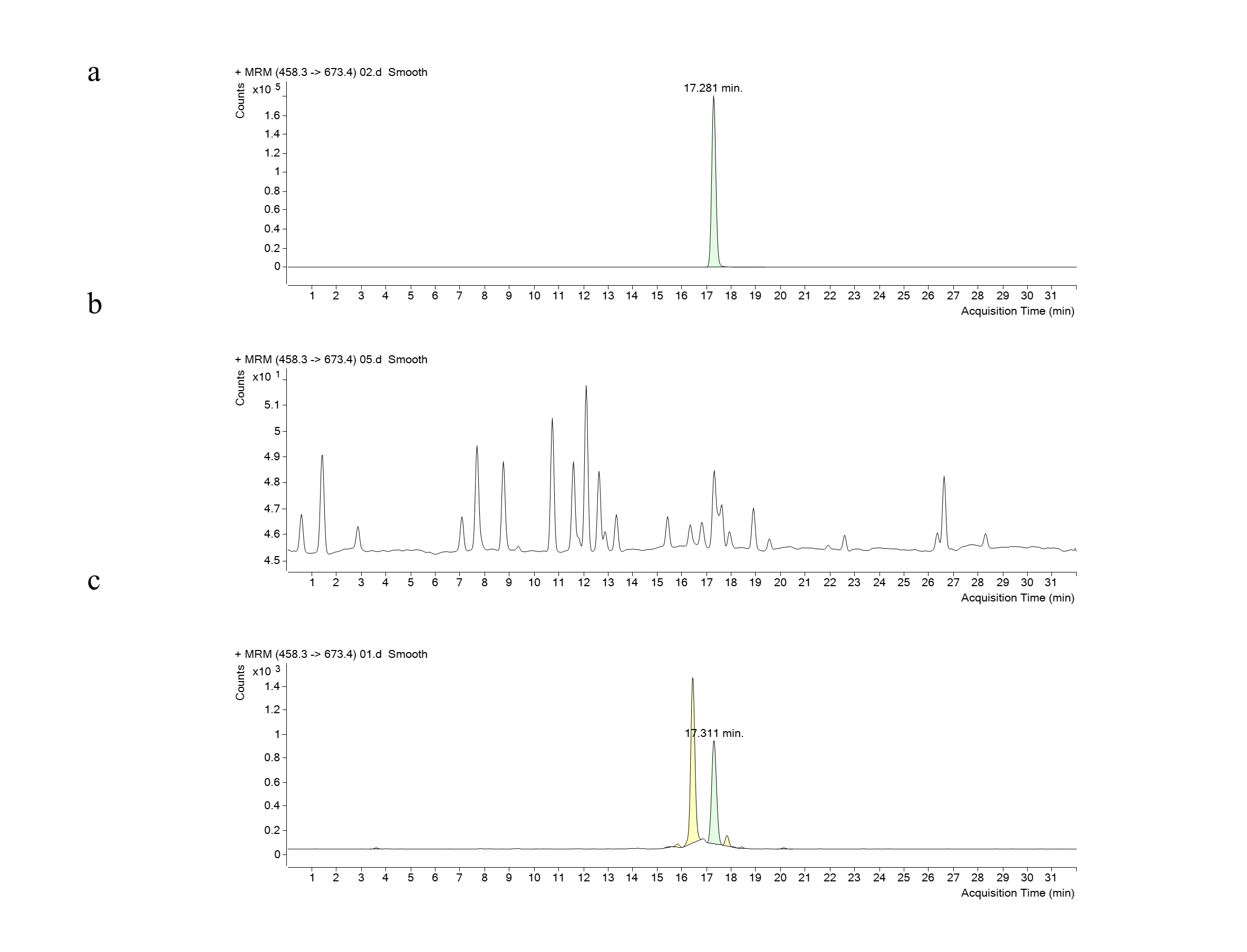

Supplement: S5 Fig — (a) TIC of pure TV910 indicates the MRM 458.3 > 673.4 for the peptide IENVILSK and the RT of 17.281 min. (b) TIV of blank (only PBSS) does not show any high-intensity peak at the specific MRM and RT. (c) TIC of the spent medium at the specific MRM and RT is indicative of the presence of the peptide of TV910 in the secreted medium of Trichomonas. (TIF) [file pntd.0006493.s005.tif]

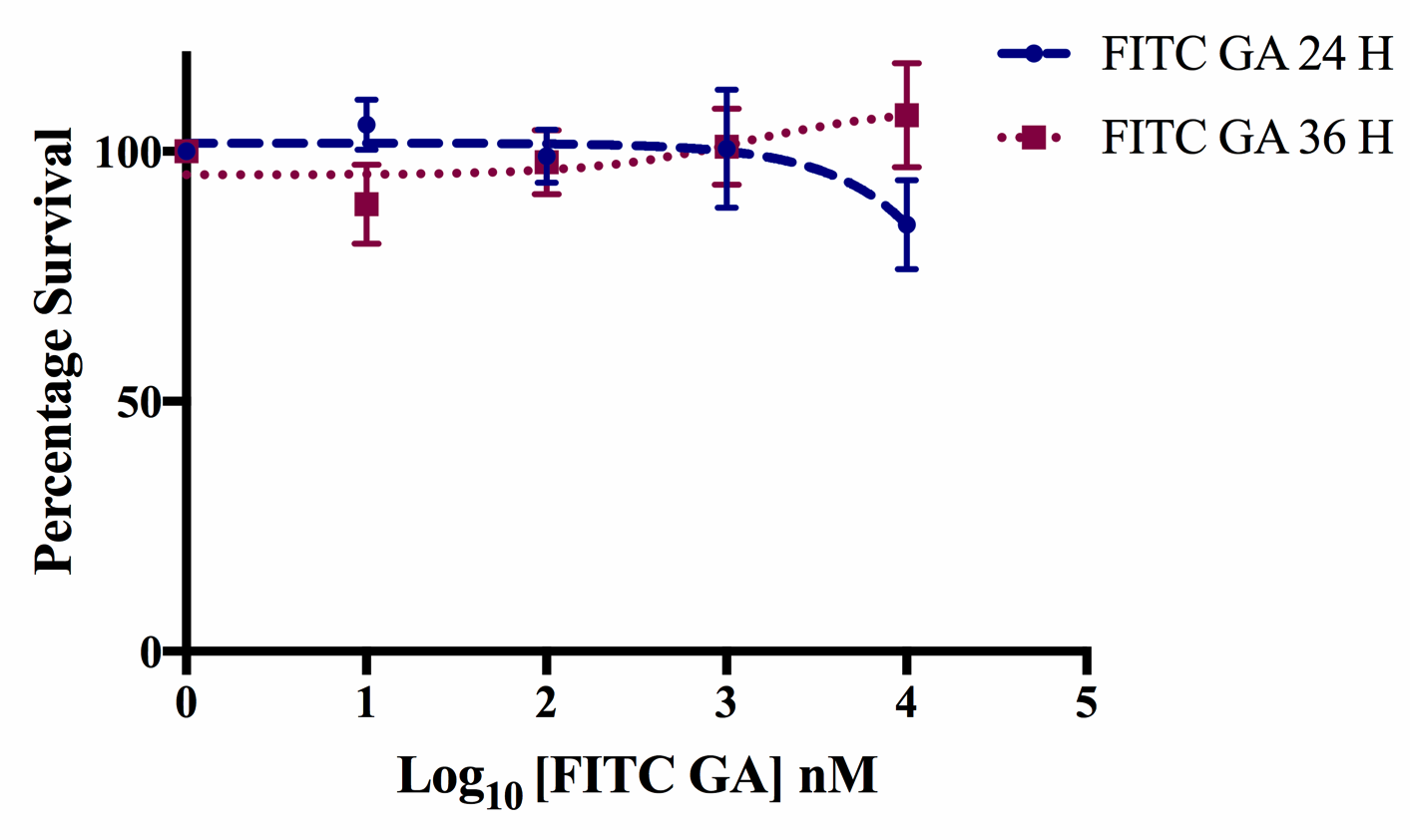

Supplement: S6 Fig — Post 24- and 36-hrs, cell survival, and viability were measured. Inhibition of extracellular Hsp90 did not significantly compromise cell survival and viability. (TIFF) [file pntd.0006493.s006.tiff]
