# Supplementary material for: A secreted Heat shock protein 90 of Trichomonas vaginalis
Source: PLoS Negl Trop Dis. 2018 May 16;12(5):e0006493. doi: 10.1371/journal.pntd.0006493 (PMC5973626; doi:10.1371/journal.pntd.0006493)
Supplement: S1 Table — (DOCX) [file pntd.0006493.s007.docx]

Supporting Information

Table S1: Analysis of *Trichomonas vaginalis* proteins which contain an ER retention motif at their C-terminus

| **Total number of annotated genes analysed*** | **97475** |
| --- | --- |
| **No. of genes with an ER retention motif** | **171** |
|  | |
| **ER Retention motif variant** | **Gene IDs for genes with an ER retention motif at the C-terminus** |
| DEL (n=74) | TVAG_120560  TVAG_076180  TVAG_379250  TVAG_456500  TVAG_192380  TVAG_193010  TVAG_193220  TVAG_021080  TVAG_365510  TVAG_402170  TVAG_494300  TVAG_319950  TVAG_150230  TVAG_165600  TVAG_209180  TVAG_206000  TVAG_424590  TVAG_401230  TVAG_363610  TVAG_269260  TVAG_403760  TVAG_244910  TVAG_474100  TVAG_390610  TVAG_040800  TVAG_174100  TVAG_030490  TVAG_157910  TVAG_315140  TVAG_470060  TVAG_381410  TVAG_455100  TVAG_473610  TVAG_406420  TVAG_233410  TVAG_164900  TVAG_472680  TVAG_144700  TVAG_143820  TVAG_RG_DS114392_5  TVAG_320510  TVAG_542450  TVAG_RG_DS116243_1  TVAG_107470  TVAG_097070  TVAG_489040  TVAG_RG_DS118205_1  TVAG_RG_DS119172_1  TVAG_RG_DS119711_1  TVAG_RG_DS119768_1  TVAG_RG_DS119893_1  TVAG_RG_DS120335_1  TVAG_524320  TVAG_RG_DS122458_1  TVAG_425710  TVAG_293720  TVAG_231110  TVAG_RG_DS124397_1  TVAG_075080  TVAG_RG_DS126936_2  TVAG_RG_DS127298_1  TVAG_RG_DS127652_1  TVAG_RG_DS128536_1  TVAG_RG_DS129835_1  TVAG_RG_DS129945_1  TVAG_RG_DS130573_2  TVAG_352090  TVAG_529480  TVAG_554850  TVAG_RG_DS138458_1  TVAG_RG_DS138611_1  TVAG_572310  TVAG_RG_DS141295_2  TVAG_RG_DS146021_1 |
| EEL(n=47) | TVAG_072080  TVAG_093700  TVAG_110080  TVAG_062500  TVAG_TEG_DS113239_3_24  TVAG_261980  TVAG_380070  TVAG_256050  TVAG_351290  TVAG_221890  TVAG_301100  TVAG_483660  TVAG_310380  TVAG_192060  TVAG_337640  TVAG_395490  TVAG_493290  TVAG_231350  TVAG_290900  TVAG_411920  TVAG_116900  TVAG_299540  TVAG_140960  TVAG_205340  TVAG_031260  TVAG_315780  TVAG_132210  TVAG_495020  TVAG_375890  TVAG_363490  TVAG_116450  TVAG_115040  TVAG_215490  TVAG_131750  TVAG_215590  TVAG_115970  TVAG_RG_DS117049_1  TVAG_042490  TVAG_404490  TVAG_RG_DS124039_1  TVAG_503410  TVAG_253330  TVAG_336470  TVAG_RG_DS135143_1  TVAG_RG_DS139600_1  TVAG_RG_DS145544_1  TVAG_RG_DS149928_1 |
| EDL (n=25) | TVAG_076650  TVAG_013540  TVAG_187780  TVAG_164300  TVAG_117450  TVAG_106560  TVAG_226470  TVAG_357980  TVAG_272460  TVAG_370420  TVAG_336590  TVAG_395660  TVAG_300330  TVAG_TEG_DS114298_1_9  TVAG_012150  TVAG_TEG_DS114382_1_2  TVAG_450250  TVAG_289900  TVAG_347610  TVAG_RG_DS117825_1  TVAG_541540  TVAG_573030  TVAG_RG_DS135995_1  TVAG_318310  TVAG_RG_DS145250_1 |
| DDL (n=25) | TVAG_119600  TVAG_183880  TVAG_283680  TVAG_402200  TVAG_462840  TVAG_046400  TVAG_RG_DS113225_14  TVAG_RG_DS113228_25  TVAG_494340  TVAG_292060  TVAG_303630  TVAG_262390  TVAG_RG_DS113248_19  TVAG_043990  TVAG_259380  TVAG_139480  TVAG_052070  TVAG_092490  TVAG_047250  TVAG_334430  TVAG_240230  TVAG_442010  TVAG_457940  TVAG_310450  TVAG_TEG_DS151134_1_1 |

* Database used: Release 36 from TrichDB “TrichDB-36_TvaginalisG3_AnnotatedProteins.fasta (http://trichdb.org/common/downloads/release-36/TvaginalisG3/fasta/data/)
