# Supplementary material for: A secreted Heat shock protein 90 of Trichomonas vaginalis
Source: PLoS Negl Trop Dis. 2018 May 16;12(5):e0006493. doi: 10.1371/journal.pntd.0006493 (PMC5973626; doi:10.1371/journal.pntd.0006493)
Supplement: S2 Table — (DOCX) [file pntd.0006493.s008.docx]

Supporting Information

Table S2:

| Peptide | Precursor Ion (+2 Charge) | Product Ion (+1 Charge) | Fragmentor | Collision Energy | Cell Accelerator Voltage | Polarity |
| --- | --- | --- | --- | --- | --- | --- |
| DELINNLGGIAK | 628.84 | 786.44 (y8) | 130 | 20.5 | 7 | Positive |
| IENVILSK | 458.27 | 673.42(y6) | 130 | 15.2 | 7 | Positive |
