# Supplementary material for: A secreted Heat shock protein 90 of Trichomonas vaginalis
Source: PLoS Negl Trop Dis. 2018 May 16;12(5):e0006493. doi: 10.1371/journal.pntd.0006493 (PMC5973626; doi:10.1371/journal.pntd.0006493)
Supplement: S3 Table — (DOCX) [file pntd.0006493.s009.docx]

Supporting Information

Table S3: Chromatographic conditions:

| **Time (Min)** | **Mobile Phase B** | **Mobile Phase A** |
| --- | --- | --- |
| 0 | 5 | 95 |
| 5 | 10 | 90 |
| 15 | 55 | 45 |
| 24 | 75 | 25 |
| 26 | 95 | 5 |
| 28 | 5 | 95 |
| 32 | 5 | 95 |
